# Chronic Bright's Disease

**Published:** 1890-09-20

**Authors:** 


### THE PRACTITIONER'S MIRROR AND RETROSPECT.

[*The Editor will be glad to receive offers of co-operation and contributions from members of the profession. There is no doubt that much valuable material full of interest and instruction is at present lost to science. This is largely so in the case of* (1) *the younger and less-known members of the hospital staff* (2) *those connected with cottage hospitals, and* (3) *the great body of medical practitioners not attached to any institution. The co-operation of all is cordially invited to enable the Editor to make* THE HOSPITAL *of the utmost possible use and value to the profession. Specialists willing to review books on their special subjects are invited to communicate this fact at once. All letters should be addressed to* THE EDITOR, THE LODGE, PORCHESTER SQUARE, LONDON, W.]

### MEDICINE.

### CHRONIC BRIGHT'S DISEASE.

Dr. Grainger Stewart, president of the Royal College of Physicians of Edinburgh, contributed a lengthy paper to the proceedings at the Berlin International Congress, entitled, "The Treatment of Chronic Bright's Disease." The author includes under the term "Bright's disease," all those forms of organic kidney affection which belong to the group first defined by Bright, including, therefore, diseases of the tubules, of the Malpighian bodies and stroma, as well as of the blood vessels, the parenchymatous and the interstitial nephritis or cirrhosis, and the waxy degeneration; "for all these individually, and associated with one another, are referable to Bright's disease." And Dr. Grainger Stewart considers a case chronic when it has lasted more than three months.

"Exposure to cold and damp, or to sudden changes of temperature," Dr. Stewart says, "is a fruitful cause not only of the initiation, but of the prolongation of kidney disease." Much advantage, he remarks, flows from residence in warm climates, and scrupulous avoidance of exposure to cold. "The clothing of patients ought always to be such as to protect from cold and to favour the action of the skin. Woollen underclothing shonld be regularly used, and should be changed whenever it is damp from perspiration, and light warm wraps should be carried about so as to be thrown on if occasion arises. It is specially important to see that the loins are well protected, and a flannel band should be worn round that part of the body." Now all this is very excellent grandmotherly advice. But it may be questioned if it were necessary from the President of the Royal College of Physicians of Edinburgh, to such an assembly of medical men as composed the medical section of the International Berlin Congress! Similarly with regard to alcohol. Dr. Stewart told his audience that, "among the causes of Bright's disease, few are more important than excessive indulgence in alcohol, and few tend more to keep up renal irritation when it has been established," an observation which might have been copied from any text-book. "With regard to the climate suitable for cases of chronic Bright's disease, Dr. Stewart advises residence in Southern Europe, in Algiers, or in Egypt, a voyage to the Cape and a sojourn there, or a similar trip to Australia or New Zealand, or to some of the West Indian islands, or to India. Now we doubt whether Southern Europe, especially the Riviera, is a suitable climate for such cases, for with the exception of perhaps Pau and Monte Carlo, the so-called "health resorts" of Southern Europe are liable to great and sudden changes of temperature, from winds peculiar to the locality, and from icy blasts from distant snow mountains. And to Algiers itself we would not send a patient of any description, for the sanitation of Algiers and its hotels is disgraceful. It is true that invalids would have a better chance on the neighbouring Sahel Hills, at Mustapha Superieur. But this involves the expense of a villa residence, and there is often a difficulty about servants. To Egypt, Australia, or New Zealand we have no objection—preferring Egypt. As to the tropics, either the West Indies or India, we say at once that the tropics are not suited for cases of chronic Bright's disease. The most recent author on tropical maladies writes: "Albuminuria should prohibit tropical residence. . . . There are in the tropics other predisposing influences in the presence of what we are accustomed to term malarious, added to those fertile causes of albuminuria to which persons are subject in England. . . . If in Europe a person has suffered from albuminuria, the recurrence of the renal affection under the tropical influences termed malarious, is most probable. . . . Experience has convinced me that tropical countries should be prohibited to all who have passed albumen, unless an accidental occurrence depending upon temporary cold or dietetic error." Although recommending change to India, Dr. Grainger Stewart observes that he "has seen a good many cases in which malarious influences had to do with renal disease." There are few countries where these so-called malarious influences are more potent than in India, and there are few countries in which sudden changes of temperature are more injuriously felt. Then there is always the chance of an attack of bad diarrhœa or cholera, the congestion of the kidneys occurring during such maladies being certain to aggravate renal disease. Prolonged residence is also liable to produce anæmia, of which albuminuria is often a complication.

Dr. Stewart is lengthy on the dietetic treatment of the disease, and he rightly says it is possible to induce a degree of albuminuria by administering a specially albuminous diet. The converse also holds good. Dr. Stewart gives three diet tables, which he considers applicable. And he then remarks, "first, it is essential that the diet be of good nutritive quality, and secondly, that it be easily digested"—an observation which again might be taken from a text-book! Dr. Stewart adds that it should not be irritating to the kidneys, nor be likely to evolve chemical products of an irritating character. Milk, the author says, meets these indications better than anything else. But there are many persons who cannot or who will not take milk, and an exclusively milk diet may excite catarrh of the stomach with functional disturbances of the liver and constipation. It should be recollected that although milk is a fluid out of the body, it becomes a solid in the stomach or intestines. Again, pure milk will sometimes disagree, but when diluted with pure water, or with lime water, will prove beneficial. Milk, Dr. Stewart thinks, is of the greatest value when there is much blocking of the tubules and dropsy on account of the diuretic properties which Dr. Stewart says milk possesses. Dr. Stewart, however, confesses that pure milk diet has not brought out the best results; but "rather a diet composed largely of milk with some pudding, fruit, and a little white meat." Now, we venture to observe that it is scarcely possible to lay down a diet which will suit all persons. There are many peculiar idiosyncracies, and what agrees well with one person will not agree with another. As it is in health so it is in disease. Only general principles of diet can be satisfactorily laid down. If more is attempted, the result is frequently disappointment of the physician and disgust of the patient. With reference to stimulants, Dr. Stewart remarks, "it is bestto avoid the habitual use of those that tend to irritate the kidneys, and in particular of gin"! "If given, such stimulants as gin, whisky, or brandy must always be well diluted! But, on the whole, wines, and especially the sweet and heavy varieties, should be avoided"!

Dr. Stewart asks the question, "Do we possess any medicine which has the power of restraining the discharge of albumen?" The only two which have proved useful are tincture of perchloride of iron and hydrochlorate of rosaniline-fuchsin, but in the majority of cases these also fail. Dropsy is an important symptom of disease of the tubules. The patient should rest in bed, should be put upon a milk diet with abundance of diluent drinks, keeping up the action of the skin by diaphoretics. Constipation should not be permitted. "But the main reliance is to be put on diuretics." Uræmia is a common cause of death. It depends on the retention of waste products which the kidneys fail to eliminate. But the author is confident that many cases of so-called uræmia are due to alteration of the circulation and of the nutrition of the brain substance, comprising minute hæmorrhages and degenerative changes. In such conditions treatment is of little service. For uræmia proper the best treatment is by acting freely on the skin and bowels. Inflammation of the gastro-intestinal mucous membrane, pleurisy and pericarditis, increased vascular tension, are other conditions referred to by the author. Had Dr. Grainger Stewart cut down his paper to less than half the length, omitting much grandmotherly advice, it would have been better suited to his audience. As it is, the paper reads very like a clinical lecture to medical students.

